# Submacular Parasite Masquerading as Posterior Pole Granuloma

**DOI:** 10.1155/2015/910383

**Published:** 2015-06-09

**Authors:** Jatinder Singh, Rajbir Singh

**Affiliations:** ^1^Aravind Eye Hospital & Postgraduate Institute of Ophthalmology, 1 Anna Nagar, Madurai, Tamil Nadu 625020, India; ^2^S.B. Dr. Sohan Singh Eye Hospital, Katra Sher Singh, Chowk Farid, Amritsar, Punjab 143001, India

## Abstract

Parasites enter the eye through hematogenous spread. The interaction with host immune system may result in its destruction but not without collateral damage to the vital retinal structures. Currently, the accepted treatment for ocular parasitosis is surgical removal or direct laser photocoagulation. A 24-year-old Indian woman presented with abrupt painless loss of vision to 5/300. A large yellow-white lesion centered at macula was observed with associated retinal and subretinal hemorrhage and neurosensory retinal detachment. A parasite was seen protruding at the center of the lesion. Fluorescein angiography demonstrated disc leakage and vessel wall staining. Ultrasonography demonstrated a highly reflective subretinal lesion with aftershadowing. Serological test was positive for anti-cysticercus (IgM) antibody. Treatment with prednisolone and albendazole resulted in resolution of the lesion within 2 months with improvement of visual acuity to 20/400. A noncystic form of subretinal cysticercosis is likely with suggestive B-scan ultrasonography and serological investigations.

## 1. Introduction

Parasites transit the vascular tunic of eye prior to entering the subretinal or intraocular space. Host immune response triggered by the parasite antigens may incite inflammation of the ocular structures. According to Lech, “to leave the parasite in the eye is to condemn the patient to blindness or total loss of the eye” [1]. The treatment advocated is urgent surgical removal or direct laser photocoagulation of the live parasite. Surgical removal of parasite from the subretinal space is complex and would necessitate performing a retinotomy. Laser photocoagulation needs precision and can be complicated by increased inflammation and development of scotoma when placed in the macula. Antihelminthic therapy for intraocular parasite carries risk of systemic adverse effects, limited effectiveness, and exacerbation of intraocular inflammation. Prior or concurrent administration of corticosteroids may help reduce the worsening of inflammation induced by the death of parasite. Monitoring of liver function and blood counts is essential if long-term antihelminthic therapy is contemplated.

We report a case of submacular live parasite masquerading as posterior pole granuloma treated with systemic antihelminthics and corticosteroids.

## 2. Case Description

**Figure 1:**
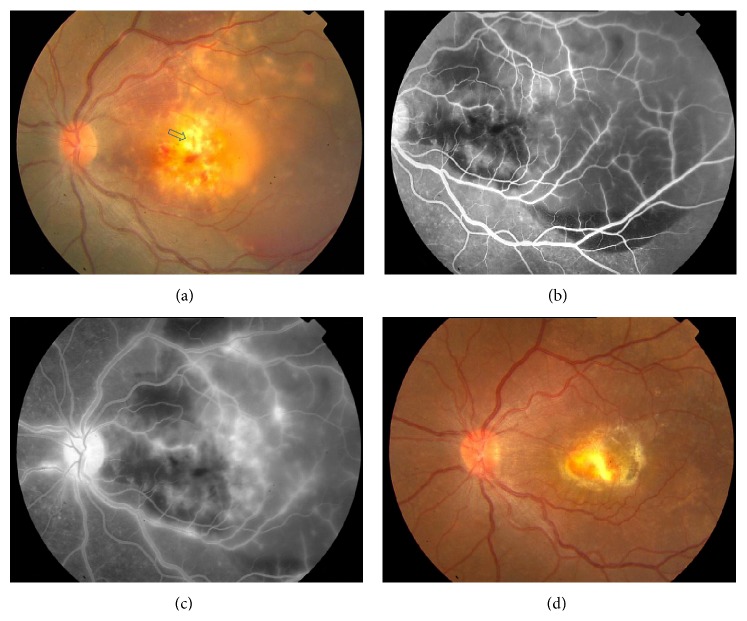
(a) Color fundus photograph showing yellow-white submacular granuloma. Also observed are satellite lesions, retinal hemorrhage, perivascular sheathing, and serosanguineous detachment of neurosensory retina extending beyond temporal vascular arcades. The arrowhead indicates the site of observed motile parasite. (b) Fluorescein angiography (early phase) demonstrates patchy blocked fluorescence by subretinal blood. (c) Late phase of fluorescein angiogram shows disc and retinal vascular leakage at and around posterior pole. (d) Color photograph documents resolution of the granuloma with resultant pigmented macular scar. The status remained stable till 4 years following treatment with albendazole and oral corticosteroids.

**Figure 2:**
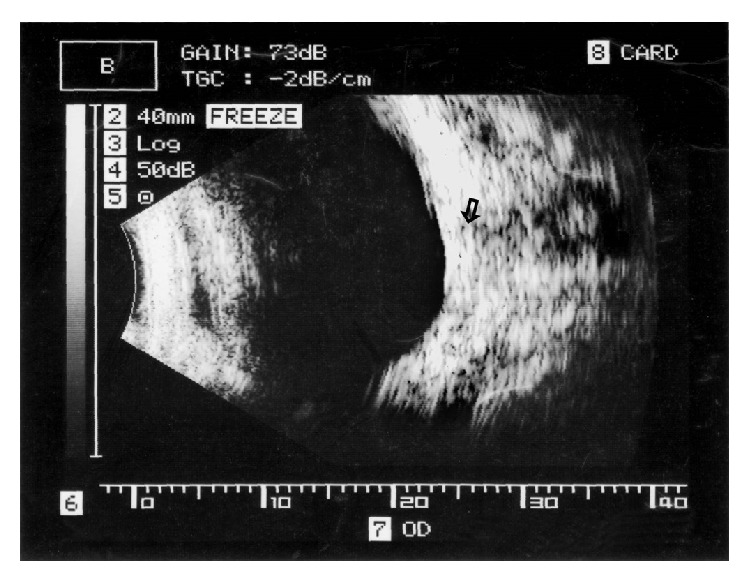
Vertical macula B-scan ultrasonogram demonstrating a calcific subretinal focus (arrow) with acoustic aftershadow of retrobulbar fat.

A 24-year-old Indian woman presented with abrupt loss of vision in the right eye of 1-week duration. Best-corrected visual acuity was 5/300 OD and 20/20 OS. The swinging flash light test demonstrated a positive relative afferent pupillary defect in the right eye. Slit lamp biomicroscopy revealed 3+ cells in the anterior vitreous. Intraocular pressure was in the normal range. A yellow-white subretinal lesion larger than 6-disc area size with adjacent satellite lesions, retinal and subretinal hemorrhage, and a serosanguineous detachment of neurosensory retina was observed in the posterior pole (Figure 1(a)). There was associated disc hyperemia and sheathing of the retinal vessels. A motile larval parasite was seen protruding from the center of the lesion. The left eye was normal. Fluorescein angiography demonstrated leakage from the disc and the retinal vessels (Figures 1(b) and 1(c)). Ultrasonography demonstrated a focal highly reflective subretinal lesion with aftershadowing (Figure 2). The magnetic resonance imaging study of the brain was normal. Blood counts were normal. Microscopic examination of stool sample did not demonstrate any evidence of parasites. Serological tests using enzyme-linked immunosorbent assay (ELISA) was negative for* Toxoplasma* and* Toxocara* but positive for anti-cysticercus (IgM) antibody.

Treatment was initiated with prednisolone 60 mg per day to be tapered by 10 mg per day every week. Albendazole 400 mg twice daily for 2 weeks was advised to be started from day 3 of therapy. Ten days following the instituted therapy, the subretinal lesion appeared to be organized with reduced subretinal fluid and associated vasculitis (supplementary figure file in Supplementary Material available online at http://dx.doi.org/10.1155/2015/910383). A dense white (opaque) plaque-like lesion located at the site of motile parasite was observed with no activity indicating the response to albendazole therapy (Figure 1(d)). The uveitis remained quiescent and the patient's vision improved to 20/400 over the next 2 months. The status remained unchanged over 4 years of follow-up.

## 3. Discussion

Our region is endemic to some parasites particularly cysticercosis [2]. Cysticercus larva may rarely present without its characteristic cystic form. Nainiwal et al. have reported with histopathological confirmation of a free-floating live cysticercus larva removed from the vitreous cavity [3]. Wender et al. reported 8 (36.4%) eyes of patients who presented with ruptured cyst; four of them were located in the subretinal space [4]. It is possible that the disruption of cyst wall and extrusion of its contents induced severe granulomatous reaction in our patient. The ultrasonographic appearance of calcification in our case supports a possible diagnosis of subretinal cysticercus larva. The lack of fluid-filled sac however did not provide the classic sonographic demonstration. Inflammation induced by the cystic contents of cysticercosis may be severe. Agarwal et al. reported a case of intraocular cysticercosis simulating retinoblastoma in a 5-year-old child [5]. The lack of neuroradiological and histopathological evidence fails to corroborate the diagnosis of a subretinal cysticercus larva. The importance of serological investigation in an endemic region for a disease is debatable [6]. Moreover, the prohibitive cost and limited utility as a guide for treatment restrict its general use. In our case, the result of serological tests provided some corroborative evidence. Surgical removal of cyst in toto is the accepted treatment for intraocular cysticercosis. However, in our case the atypical manifestation and submacular location precluded a safe removal of the parasite. The specific location of the parasite at the macula amidst subretinal exudates in an inflamed eye limits the options of surgical and laser therapy. Lim and Chee successfully treated a subretinal cysticercosis with use of albendazole coupled with immunosuppressive dose of oral steroids [7]. Our case illustrates that a high index of suspicion in a patient from endemic region, a detailed examination under high magnification, and investigations may provide a clue to the diagnosis. The management with albendazole and oral corticosteroids helped in abolishing the parasite in our patient's eye without any further complication.

## Supplementary Material

Color fundus picture of the left eye at 10 days (a), 4 weeks (b) and 8 weeks (c) after initiation of medical therapy. Note the regression of the subretinal hemorrhage, perivascular sheathing, disc edema and consolidation of the posterior pole granuloma. Bright yellow-white center of the granuloma appeared calcified; the same was demonstrated on B-scan ultrasonography.
