# Supplementary material for: Submacular Parasite Masquerading as Posterior Pole Granuloma
Source: Case Rep Ophthalmol Med. 2015 Jun 9;2015:910383. doi: 10.1155/2015/910383 (PMC4477082; doi:10.1155/2015/910383)
Supplement: Supplementary file 1 — Color fundus picture of the left eye at 10 days (a), 4 weeks (b) and 8 weeks (c) after initiation of medical therapy. Note the regression of the subretinal hemorrhage, perivascular sheathing, disc edema and consolidation of the posterior pole granuloma. Bright yellow-white center of the granuloma appeared calcified; the same was demonstrated on B-scan ultrasonography. [file 910383.f1.pdf]

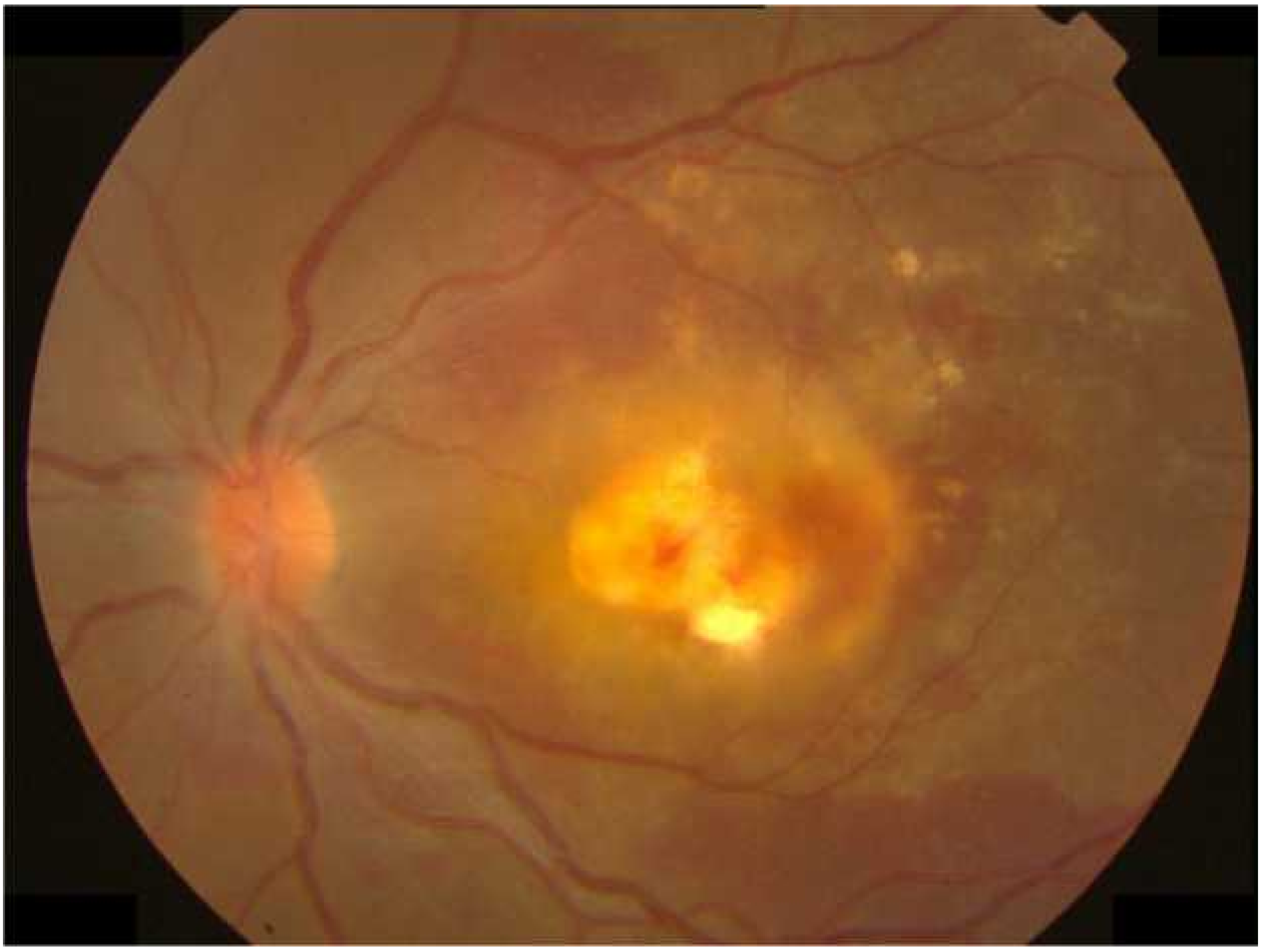

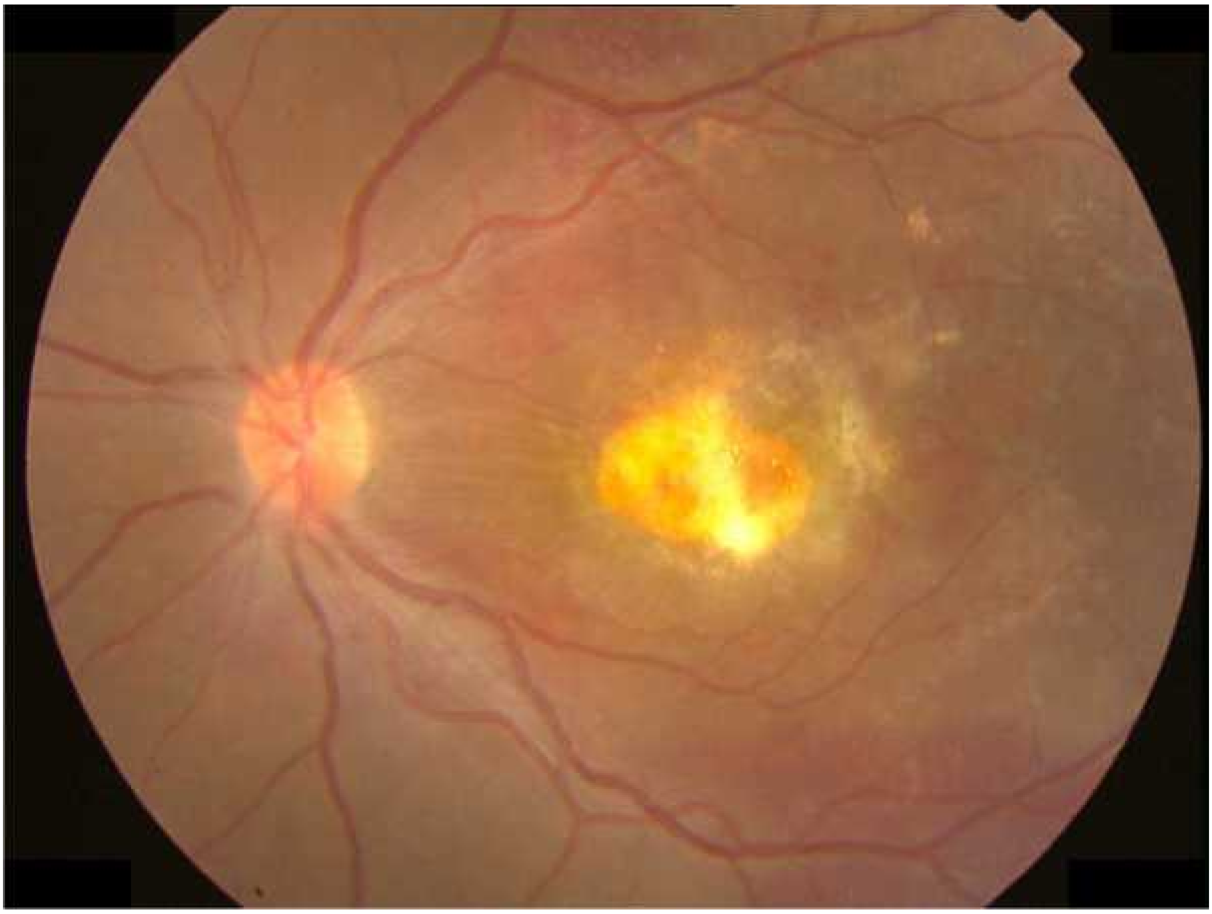

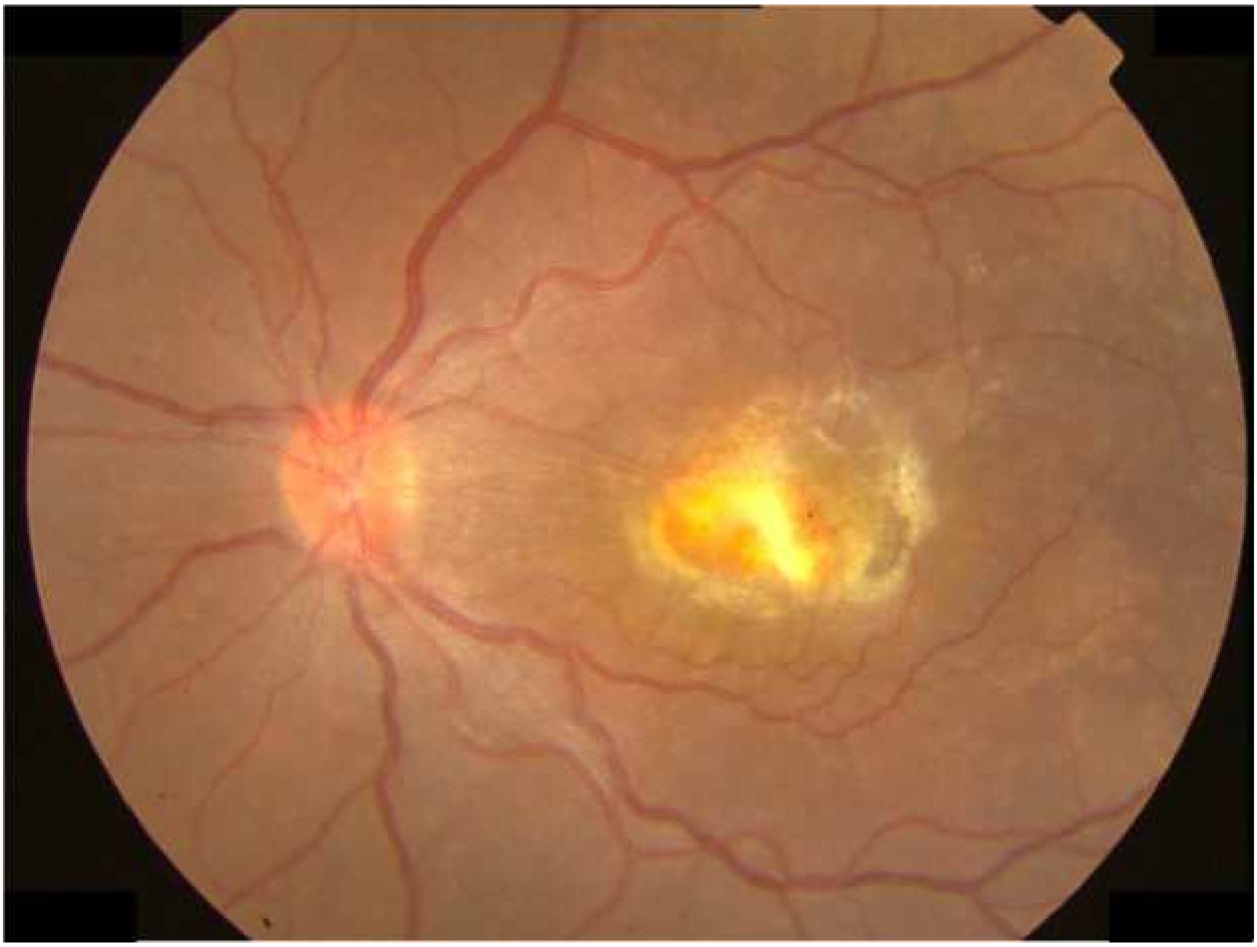

Color fundus picture of posterior pole (Left eye) at 10 days (A), 4 weeks (B) and 8weeks (C) follow-up after initiation of medical therapy. Note the regression of the subretinal haemorrhage, perivascular sheathing, disc edema and consolidation of the posterior pole granuloma. Bright yellow-white center of the granuloma appeared calcified, the same was demonstrated on B-scan ultrasonography.
